# Prevalence and income-related equity in hypertension in rural China from 1991 to 2011: differences between self-reported and tested measures

**DOI:** 10.1186/s12913-019-4289-5

**Published:** 2019-07-01

**Authors:** Dan Cao, Zhongliang Zhou, Yafei Si, Xiao Xiao, Xiao Wang, Chi Shen, Yangling Ren, Min Su, Shuyi He, Jianmin Gao

**Affiliations:** 1 School of Public Policy and Administration, Xi’an Jiaotong University, Xi’an, People’s Republic of China; 2 International Business School Suzhou, Xi’an Jiaotong-Liverpool University, Suzhou, People’s Republic of China

**Keywords:** Tested hypertension, Self-reported hypertension, Equity, Concentration index

## Abstract

**Background:**

Along with economic growth and living standard improvement, hypertension has become one of the most prevalent chronic diseases in China. Self-reported measures and tested measures of hypertension may differ significantly due to the low awareness of prevalence. The objective of this study is to figure out whether and how self-reported measures differ from tested measures in terms of prevalence and equity.

**Method:**

We have used data from the China Health and Nutrition Survey database from 1991 to 2011 and extracted the data of rural areas using *hukou* system. Hypertension is categorized into two groups: self-reported hypertension and tested hypertension. To evaluate the equity of self-reported hypertension and tested hypertension, we calculated their Concentration Index (C) and decomposed C based on which we have obtained the horizontal-inequity index (HI) of each year. Probit Model was deployed to analyze the key determinants of hypertension prevalence.

**Results:**

We found that the prevalence of both self-reported hypertension and tested hypertension have sharply increased from 1991 to 2011 in rural China and the population of tested hypertension was significantly larger than that of self-reported hypertension. For self-reported hypertension, prevalence rate increased from 2.72 to 13.2% and for tested hypertension it increased from 11.01 to 25.05%. Both of the Concentration Index (C) and horizontal-inequity index (HI) of self-reported hypertension and tested hypertension appeared to be contradictory. The C and HI of self-reported hypertension in 2011 were 0.032 and 0.060 respectively while the C and HI of tested hypertension were − 0.024 and − 0.015 respectively.

**Conclusion:**

More efforts should be put into for improving the poor’s health, especially in equal access to health services. Symptom-based measures such as tested hypertension should be adopted more widely in empirical studies.

**Electronic supplementary material:**

The online version of this article (10.1186/s12913-019-4289-5) contains supplementary material, which is available to authorized users.

## Background

Chronic diseases, such as cardiovascular and cerebrovascular diseases, are becoming increasingly prevalent [1] and hypertension is one of the most prevalent but preventable one amongst them [2]. The number of adults with hypertension in 2025 is predicted to be 1.56 billion and the total number of them in developing countries is substantially higher than developed countries [3]. Despite of the great economic growth, since the reform and opening-up policy was implemented, one of the most alarming issue is that the morbidity rate of hypertension has increased from 1.19% in 2003 to 9.89% in 2013 [4, 5]. Although prior studies have proved that a substantial health inequity exists not only in China but also in other countries [6–9]. This issue in China has to be addressed rigorously since the new objective of “healthy China” has been put forward in the National Health Conference in 2016. Also, hypertension burden appeared differently in rural and urban areas in China [10, 11]. In 2012, the hypertension cases in China had reached up to 266 million [12], yet the prevalence of hypertension remains inflating, whereas the awareness, treatment and control of hypertension remained at a inadequate especially in the rural areas [13]. Several studies have stressed that the population of hypertension in rural China has increased rapidly. In the last decades, the growth of hypertension in rural China was higher than that in urban areas and the prevalence in rural areas nearly reached the same level in urban areas [14, 15]. Recently, researchers were even found that rural residents have higher hypertension prevalence than urban residents in Southwest China [16].

Socioeconomic differences in chronic disease prevalence have been found worldwide. For instance, previous studies have proved that socioeconomic inequalities exist among patients with some fatal chronic diseases, such as cancer and heart diseases [9]. A study focusing on the chronic diseases in Slovenia also suggests that the prevalence is significantly higher in the population with lower socioeconomic and employment status [17]. Moreover, the relationship between hypertension prevalence and several potentially modifiable factors such as education, profession and income level have been studied [18, 19]. Over the last decades, previous researchers have agreed on that the socioeconomic status can significantly affect hypertension prevalence and the severity of hypertension [10]. A related study also shows the same result that lower education is associated with a higher risk of pre-hypertension [20].

China has started equalizing the basic public health services since 2009, granting sufficient access to basic public health services [21]. Thus hypertension management was improved substantially from 2008 to 2012 and the inequity across regions declined over time [22]. Access to some services such as chronic diseases screening is still far away from being equalized. Regardless of significant improvement in coverage of basic public health services, more equalization needs to be improved [23]. Unbalanced access and utilization results in uneven awareness and thus it is generally recognized that wealthier people have more opportunities to become aware of the prevalence of chronic diseases. Researchers usually use two measures to evaluate hypertension prevalence: self-reported prevalence and tested prevalence. These two measures may differ a lot due to the uneven awareness of hypertension prevalence. The disparity between the poor and the rich may lead to a biased result and mislead the government to implement related policies [24]. Researchers have also found that self-reported measures can lead to significant deviation from the real prevalence and inequality, and using symptom-based measures can be an effective way to eliminate the reporting bias [25, 26].

To find out whether and how self-reported measures differ from tested measures, we conducted this study. In this study, we extracted the rural data from a national representative database-- the China Health and Nutrition Surveys from 1991 to 2011 using the *hukou* system. We deployed both of these two methods to measure hypertension: one is self-reported hypertension and the other is tested hypertension namely symptom-based. Concentration index was adopted to estimate inequity. Previous literature has addressed that unequal access to health care utilization may cause a prevalence deviation between self-reported measures and tested measures. Self-reported measure causes an underestimation of real prevalence, especially for those with low socioeconomic status. Hence, in this study, we hypothesize that both the prevalence and the equity of self-reported and tested measures differ a lot.

## Methods

### Data sources

We used a national representative database from China Health and Nutrition Surveys (CHNS). CHNS is a longitudinal survey from the late 1980s conducted by the University of North Carolina Center for Population Studies, the National Institute of Nutrition and Food Safety and the China’s Center for Disease Control. The CHNS data contains new household formation, replacement communities and households, and all household members [27]. The questionnaire contains 12 dimensions: population density, economic activity, traditional markets, modern markets, transportation infrastructure, sanitation, communications, housing, education, diversity, health infrastructure and social services.

CHNS survey covers nine provinces that vary substantially in geography, economic level, public resources and health indicators. The samples in each province were selected with a multistage, random cluster process. The counties were stratified by income (low, middle and high) in each province and a weighted sampling scheme was used to select four counties randomly. Villages and towns within the counties and urban and suburban neighborhoods within the cities were chosen randomly. Approximately there were 4400 households in the whole survey covering 19,000 individuals [28].

The CHNS contains a weakness; the follow-ups were missing some chunks of data every year. There were three major reasons: 1) missing population that couldn’t be found because of travel, hours of work or play, 2) school children who were in boarding schools, 3) migrants work for working population. But the CHNS considered loss follow-ups into their design and recruited new participants as replenishment population if there were no more than 20 households or if respondents had constituted a new family [29]. This design of replenishment sample made up the weakness caused by the loss of enrolled subjects. The cross sectional data of each wave was regarded as national representative in many other researches [30–32].

### Measures/variables

#### Dependent variables

The design of this study contains two dependent variables: tested prevalence and self-reported prevalence. For the tested prevalence, the CHNS measured respondent’s blood pressure three times and we took their average value. We established the database of tested hypertensive persons whose SBP (systolic blood pressure) were higher than 140 mmHg and their DBP (diastolic blood pressure) were higher than 90 mmHg. Self-reported hypertensive persons were classified as; who knew they were suffering from high blood pressure or taking any anti-hypertension drugs by answering the questions: “Have you ever been diagnosed with hypertension by a doctor?” or “Are you currently taking any anti-hypertensive medication?”

#### Independent variables

According to prior studies, we adopted age, gender, BMI, economic level, smoking, drinking, schooling, marital status, region and physical examinations of the past 4 weeks [33]. The economic level in this study was defined by grouping inflation to 2011 household income per capita into five cohorts: the poorest, the poorer, the middle, the richer and the richest. If the respondent has smoked even once, the person is classified as a Smoker. Also if the respondent drinks any form of alcohol for more than 3 times a week, the person is classified as Drinker. Regions are categorized into three: east, middle and west, which are consistent with the standard in Statistic Book of PRC. More details about independent variables are presented in Table 1.

**Table 1:** Independent Variables

Unavoidable variables		Avoidable variables	
Name	Explanation	Name	Explanation
Agegroup	18~45^a^ =0 46~59 = 1 60 and above =2	Economic level	The poorest^a^ = 0 The poorer = 1 The middle = 2 The richer = 3 The richest = 4
Gender	Male^a^ = 0 Female = 1	Smoking	No^a^ = 0 Yes = 1
		Drinking	Less than 3 times a week^a^ = 0 More than 3 times a week = 1
		Have physical examination in the past 4 weeks	No^a^ = 0 Yes = 1
		Region	East^a^ = 0 Middle = 1 West = 2
		Schooling	Illiteracy^a^ = 0 Primary and junior high school = 1 High and technical secondary school = 2 Junior college and above = 3
		BMI	BMI < 18.5^a^ =0 18.5 ≤ BMI < 24 = 1 24 ≤ BMI < 28 = 2 BMI ≥ 28 = 3
		Marital status	Unmarried^a^ = 0 Married = 1 Others = 2

^a^is the control group of dummy variables

All independent variables are grouped into unavoidable variables and avoidable variables. The unavoidable variables refer to factors that couldn’t be avoided in hypertension including age and gender, while avoidable variables contain economic level, smoking history, drinking history, physical examinations of the past 4 weeks, region, schooling, BMI and marital status [34, 35].

### Measure of equity

The feasibility and reliability for Concentration Index(C) and decomposition of C to measure health equity have been well documented [36, 37]. The concentration index can expose the relationship between health outcomes, such as self-reported health status and living standards like income level and wealth index. More widely, the concentration index can examine inequality not only in health outcomes but also in any health sector variable of interest [38], such as hypertension prevalence in this article. In our study, the two key variables underlying the concentration index are hypertension prevalence, the distribution of which is the subject of interest, and income level against which the distribution is to be assessed. We can see the degree of inequality of the hypertension prevalence distributes among different living standards. Further more, we decomposed the C to figure out how such inequality can be explained. The following specifically shows how we computed C and sub-section 2.4 decomposition of C.

#### Concentration index

In general, the Concentration Index (C) is considered to be a good indicator reflecting inequality in health status caused by socioeconomic factors [35, 39]. In this study, we used the concentration index to measure the inequality of hypertension prevalence of people in different income groups. The range of the concentration index is from − 1 to 1. If people with different economic levels have the same probability to suffer from hypertension, the concentration index equals to 0. If the concentration index is negative, it indicates hypertension prevalence is pro-poor and if the concentration index is positive, it indicates hypertension prevalence is pro-rich. We calculated the concentration index with the equation below:

$$ \mathrm{C}=\frac{2}{\mu}\mathit{\operatorname{cov}}\left(y,{R}_i\right) $$

Where R_i_ represents the proportion of individual i in sample sorted by economic level (inflated to 2011 per capita household income), y_i_ is hypertension prevalence, μ represents the average of hypertension prevalence.

### Measures of horizontal inequity

#### Decomposition of concentration index

Decomposition of concentration index can provide a reliable way to analyze the contribution of various factors to the inequality of hypertension by estimating each factor’s effect on hypertension prevalence using a Probit model [40]. The Probit equation is as below.

$$ \Pr \left(\mathrm{Y}=1|\mathrm{X}\right)=\varnothing \left({\mathrm{X}}^{\prime}\upbeta \right), $$

Where Pr is the probability of suffering from hypertension, ∅ represents the cumulative function of the normal distribution, β is the parameter evaluated by maximum likelihood method.

After decomposing the concentration index into the contribution of various factors to the inequality of hypertension and summing up the C’s of all avoidable variables, we obtained the horizontal inequity of hypertension prevalence, of which the unavoidable variables contained demographic variables and prevalence variables, and the avoidable variables contained economic level, risk behaviors of hypertension and other avoidable variables. In this study, we decomposed both the C’s of tested prevalence and self-reported prevalence of each year.

We estimated each factor’s effect on hypertension prevalence by the model below:

$$ {y}_i={\alpha}^m+\sum \limits_j{\beta}_j^m{x}_{ji}+\sum \limits_k{\gamma}_k^m{z}_{ki}+{\mu}_i, $$

Where *y*_*i*_ represents the dependent variable, *x*_*ji*_ represents the unavoidable variable, and *z*_*ki*_ is the avoidable variable, ${\beta}_j^m$ and ${\gamma}_k^m$ represent the partial effects, *μ*_*i*_ is the residual term.

The concentration index formula for the horizontal inequity is presented as below:

$$ C=\sum \limits_j\left({\beta}_j^m{x}_{ji}/\mu \right){C}_j+\sum \limits_k\left({\gamma}_k^m{z}_{ji}/\mu \right){C}_k+\frac{GC_k}{\mu_i}, $$

Where C represents the concentration index of hypertension prevalence, *C*_*j*_ represents the concentration index of *x*_*j*_, *C*_*k*_ is the concentration index of *z*_*k*_, *GC*_*k*_ is the concentration index of residual terms. This formula indicates that the concentration index of hypertension prevalence is obtained by adding weight-sum of avoidable variables’ and unavoidable variables’ C’s. Furthermore, the horizontal-inequity index can be measured by controlling the contribution of the unavoidable variables.

## Results

### Descriptive results of 2011

Excluding respondents under 18 and singular values we have a sample of 122,945 observations. The sample values contain 11,119 in 1991, 10,828 in 1993, 11,891 in 1997, 13,324 in 2000, 13,194 in 2004, 15,922 in 2006, 16,313 in 2009 and 19,722 in 2011 respectively. The descriptive results of our sample are presented in Table 2. A table about baseline subjects involved in 1991 and new subjects of each wave is shown in the (Additional file 1: Table S1).

**Table 2:** Descriptive results (%)

		1991	1993	1997	2000	2004	2006	2009	2011
Agegroup	18~45	66.41	65.40	61.73	58.88	54.11	59.49	56.29	51.72
	46~59	17.89	18.17	20.40	23.05	25.40	21.39	22.85	25.07
	60 and above	15.69	16.43	17.86	18.07	20.50	19.12	20.86	23.21
BMI	< 18.5	10.26	9.02	8.02	6.78	6.26	6.09	6.53	4.99
	18.5~24	71.14	70.40	65.92	60.97	57.85	56.86	53.81	50.47
	24~28	15.56	17.46	20.95	25.42	27.73	28.69	30.04	32.49
	> 28	3.04	3.13	510	6.84	8.06	8.36	9.62	12.05
Schooling	Illiterate	24.03	21.90	18.11	16.37	12.26	14.36	12.50	9.85
	Primary or junior high school	60.11	61.60	62.41	62.50	62.87	58.20	61.98	57.20
	High school or technical secondary school	13.39	13.91	16.54	17.19	19.97	20.89	19.32	22.66
	Junior college and above	2.47	2.59	2.94	3.93	4.91	6.54	6.20	10.29
Marital status	Unmarried	18.37	18.37	18.33	17.92	9.16	7.42	6.84	5.96
	Married	75.46	74.61	74.58	75.09	82.06	83.94	82.94	83.64
	Others	6.76	7.01	7.09	6.99	8.78	10.23	10.23	10.40
Region	West	33.67	32.86	24.01	32.24	31.16	30.84	29.87	36.62
	Middle	52.34	52.68	61.37	55.04	56.31	55.26	56.72	46.75
	East	13.99	14.46	14.62	12.73	12.54	13.90	13.41	16.63
Gender	Male	48.63	48.84	48.97	48.86	48.94	47.26	46.74	47.00
	Female	51.37	51.16	51.03	51.14	51.06	52.74	53.26	53.00
Smoking	Yes	35.07	33.98	31.90	31.52	32.58	31.53	31.35	30.66
	No	64.93	66.02	68.10	68.48	67.42	68.47	68.65	69.34
Drinking	Yes	37.86	35.61	35.99	35.24	32.90	31.89	33.25	33.92
	No	62.14	64.39	64.01	64.75	67.10	68.11	66.75	66.08
Physical examination	Yes	1.03	0.92	0.91	0.83	3.44	3.36	4.00	7.26
	No	98.97	99.08	99.09	99.17	96.56	96.64	96.00	92.74

### Comparison of prevalence rate

**Fig. 1:**
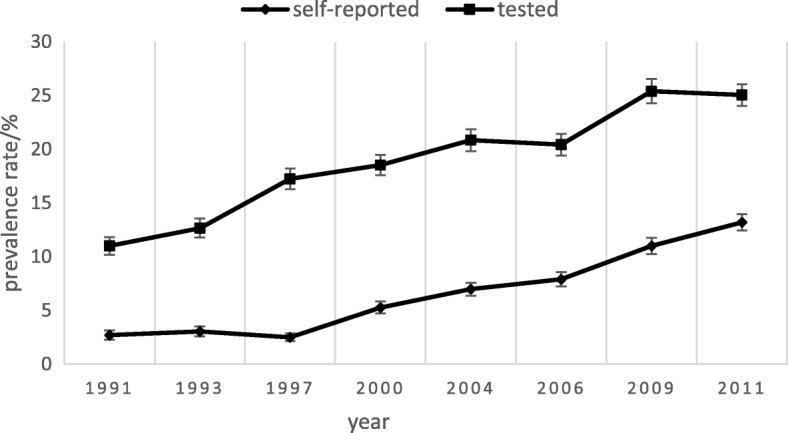
The prevalence of self-reported hypertension and tested hypertension

Figure 1 displays the prevalence rate of self-reported hypertension and tested hypertension, which suggests that the prevalence rate of self-reported hypertension in rural China has been increasing from 1991 (2.72%) to 2011 (13.2%). The prevalence of tested hypertension in rural China also has increased from 11.01% in 1991 to 25.05% in 2011. The increasing trend of self-reported prevalence and tested prevalence appears consistent. Figure 1 also indicates that the morbidity rate of self-reported hypertension increased more rapidly after 2000.

Considering the age of follow-ups would increase across the time, this study also evaluated the age-adjusted prevalence of both self-reported and tested hypertension. Both the prevalence’s, self-reported and tested hypertension, was adjusted to age distribution of the corresponding year in order correct the prevalence deviation caused by the follow-up getting older across time. The results of age-adjusted prevalence are placed in the [Additional file 2: Figure S1]. Additionally, to guarantee the hypertension screening in this survey would not affect the self-reported prevalence, identified baseline subjects and new subjects of each year and conducted Chi-Squared test. The null hypothesis was that the respondent’s self-reported hypertension status was independent of the respondent being in baseline population or new population. The results of Chi-Squared test suggested that the prevalence of two kinds of respondents varied in some years, but had no significant difference in most years, which is shown in the [Additional file 3: Table S2]. The results confirmed that hypertension screening across time has little effect on self-reported hypertension prevalence.

### Comparison of equity

#### Concentration index

In this study, we used the concentration index to measure the inequality of hypertension prevalence of people with different income groups. The concentration indexes of self-reported hypertension from 1991 to 2011 are presented in Table 3.

**Table 3:** Concentration Index from 1991 to 2011

Year	Self-reported hypertension			Tested hypertension		
	*C*	95%CI		*C*	95%CI	
1991	0.118	0.023	0.213	0.088	0.044	0.132
1993	0.144	0.055	0.232	−0.003	− 0.043	0.038
1997	0.064	−0.020	0.148	−0.008	−0.040	0.025
2000	0.052	−0.008	0.112	0.010	−0.020	0.040
2004	0.110	0.058	0.161	0.031	0.002	0.059
2006	0.119	0.071	0.168	−0.030	−0.058	−0.001
2009	0.065	0.026	0.104	0.006	−0.020	0.032
2011	0.032	−0.002	0.065	−0.024	−0.047	− 0.00009

It is evident that the concentration indexes of self-reported hypertension from 1991 to 2011 are all positive and statistically significant in most of the years. Nonetheless, when tested hypertension is included, the concentration indexes present the opposite bias. In addition, the concentration indexes of tested hypertension for the most years indicate an opposite trend, for example, − 0.003 [95%CI (− 0.043,0.038)] in 1993, − 0.008 [95%CI (− 0.040,0.025)] in 1997, − 0.030 [95%CI (− 0.058,-0.001)] in 2006, and − 0.024 [95%CI (− 0.047,-0.00009)] in 2011. Therefore, tested hypertension is not pro-rich, instead pro-poor in these years. Table 3 also indicates that the concentration indexes are getting closer to 0 since 2009, that might be due to the start of basic public services equalization in 2009.

#### Decomposition of concentration index

Probit Model was adopted in this study to analyze the effects of independent variables on hypertension prevalence. Taking decomposition results of year 2011 in Table 4 as an example, controlling for confounding variables compared to those under 45, people with older age have more probability to have both tested hypertension and self-reported hypertension. Decomposition results of other years are presented in the [Additional file 4: Table S3-S4]. In 2011, the difference between people underweight and people with higher BMI is statistically significant suggesting that the latter are more likely to get hypertension (either based on tested hypertension or self-reported hypertension). People with higher education level have a lower probability of tested hypertension compared with people who are illiterate. The results of self-reported hypertension are not so statistically significant. Compared to unmarried, married people are more likely to suffer from hypertension. People living in Middle and West China have more probability to get hypertension. Females are less likely to suffer from tested hypertension compared to males, which is opposite to the results of self-reported hypertension but not statistically significant. Drinking is also a risk factor of tested hypertension but not self-reported hypertension. Those who had a physical examination in the past 4 weeks are more likely to have self-reported hypertension.

**Table 4:** The regression results of 2011

	Self-reported hypertension						Tested hypertension					
	*dy/dx*	*Std. Err*	Demand elasticity	C	Contribution	%	*dy/dx*	*Std. Err*	Demand elasticity	C	Contribution	%
The poor	−0.011	0.010	−0.017	− 0.400	0.007	20.9	0.002	0.016	0.001	−0.400	−0.001	2.4
The middle	0.010	0.011	0.015	0.001	0.000	0.03	0.025	0.017	0.020	0.001	0.000	−0.1
The richer	0.004	0.011	0.006	0.401	0.003	8.1	−0.024	0.016	−0.020	0.401	−0.008	34
The richest	0.008	0.011	0.012	0.801	0.010	30.4	−0.019	0.016	−0.015	0.801	−0.012	52.6
46~59	0.138^c^	0.013	0.262	0.081	0.021	67.3	0.189^c^	0.015	0.190	0.081	0.015	−66.6
60 and above	0.281^c^	0.017	0.493	−0.127	− 0.063	197.5	0.335^c^	0.017	0.312	−0.127	− 0.040	170.4
18.5 ≤ BMI < 24	0.043^b^	0.018	0.165	−0.010	−0.002	−5.2	0.120^c^	0.027	0.243	−0.010	−0.002	10.4
24 ≤ BMI < 28	0.131^c^	0.026	0.323	0.031	0.010	31.5	0.274^c^	0.033	0.357	0.031	0.011	−47.6
BMI ≥ 28	0.258^c^	0.041	0.236	0.025	0.006	18.3	0.444^c^	0.038	0.215	0.025	0.005	−22.7
Primary and junior high school	−0.005	0.010	− 0.022	− 0.028	0.001	2.0	− 0.038^b^	0.016	− 0.087	− 0.028	0.002	−10.6
High school or technical secondary school	−0.013	0.013	−0.023	0.125	−0.003	−9.1	−0.075^c^	0.018	−0.068	0.125	−0.009	36.8
Junior college and above	−0.028	0.021	−0.022	0.152	−0.003	−10.5	− 0.067^a^	0.032	− 0.028	0.152	− 0.004	18.1
Married	0.072^b^	0.020	0.453	0.016	0.007	22.6	0.047	0.031	0.159	0.016	0.003	−10.8
Other status	0.156^c^	0.072	0.123	−0.177	−0.022	−68.8	0.103^b^	0.045	0.043	−0.177	−0.008	32.6
The middle region	−0.030^c^	0.007	−0.105	−0.109	0.011	36.0	−0.033^c^	0.016	−0.061	−0.109	0.007	−28.8
The western region	−0.047^c^	0.008	−0.059	−0.147	0.009	27.4	−0.035^b^	0.014	−0.023	−0.147	0.003	−14.7
Smoking	0.005	0.010	0.010	0.008	0.000	0.3	0.006	0.014	0.007	0.008	0.001	−0.3
Female	0.013	0.010	0.052	−0.027	−0.001	−4.5	− 0.047^c^	0.014	− 0.101	−0.027	0.003	−11.9
Drinking	0.010	0.010	0.007	0.142	0.001	3.1	0.046^c^	0.016	0.017	0.142	0.002	−10.3
Having physical examination	0.102^c^	0.020	0.056	0.120	0.007	21.1	0.023	0.022	0.007	0.120	0.001	−3.4
N	7124						7003					
R^2^	0.1691						0.1357					

^a^, ^b^, ^c^: significantly different from zero at the 0.1, 0.05 and 0.01 level, respectively

According to the decomposition results of 2011, considering only one variable effect on hypertension by controlling other factors, the prevalence of hypertension will be concentrated on the rich if the contribution is positive, otherwise, the prevalence of hypertension will be concentrated on the poor. Excluding the total contribution of all variables from the concentration index of hypertension, we obtain the contribution of unexplained variables. In Table 4 it is apparent that in the rural area the prevalence of self-reported hypertension in 2011 can be explained mainly by aged 45~59(67.3%), aged 60 and above (197.5%) and other marital status (− 68.8%). While the prevalence of tested hypertension can be explained mainly by the richest (52.6%), aged 45~59 (66.6%) and aged 60 and above (170.4%).

We calculated the horizontal-inequity indexes of the two hypertension groups from 1991 to 2011. As presented in Table 5, the horizontal-inequity indexes of self-reported hypertension are positive in all 8 years and are statistically significant in most years, while the indexes of tested hypertension are negative in some years, such as − 0.004 in 1993, − 0.028 in 1997, − 0.002 in 2000, − 0.033 in 2006, − 0.009 in 2009 and − 0.015 in 2011. Although the horizontal-inequity indexes of tested hypertension are not statistically significant in some years, but they still show clear differences compared with horizontal-inequity indexes of self-reported hypertension.

**Table 5:** Horizontal-inequity of the two hypertension groups from 1991 to 2011

Year	Self-reported hypertension					Tested hypertension				
	*C*	Contribution of unavoidable variables	HI	95%CI		*C*	Contribution of unavoidable variables	HI	95%CI	
1991	0.118	0.100	0.018	− 0.007	0.113	0.088	0.020	0.068	0.024	0.112
1993	0.144	0.005	0.139	0.050	0.227	− 0.003	0.001	− 0.004	− 0.004	0.037
1997	0.064	0.009	0.055	−0.029	0.139	−0.008	0.020	− 0.028	− 0.060	0.005
2000	0.052	0.013	0.039	−0.021	0.099	0.010	0.012	−0.002	−0.032	0.028
2004	0.110	0.012	0.098	0.046	0.149	0.031	0.025	0.016	−0.023	0.034
2006	0.119	−0.024	0.143	0.095	0.192	−0.030	0.003	−0.033	−0.061	− 0.004
2009	0.065	0.008	0.057	0.018	0.096	0.006	0.015	−0.009	− 0.035	0.017
2011	0.032	−0.028	0.060	0.026	0.093	−0.024	−0.009	− 0.015	−0.038	0.009

### Sensitivity analysis

To build the confidence in our concentration index results, we excluded the results whose income level ranks the first 1% and the last 1% in our sample and calculated the concentration indexes of 8 years again. Table 6 shows the results of our sensitivity analysis. The table suggests that after exclusion of extreme data, the concentration indexes were consistent with the former results. Beyond that, the trend of 8 years in altered sample was also identical with the trend in the original sample.

**Table 6:** Concentration index of samples

Year	Self-reported hypertension		Tested hypertension	
	Altered sample	Original sample	Altered sample	Original sample
1991	0.122	0.118	0.085	0.088
1993	0.156	0.144	0.000	−0.003
1997	0.041	0.064	−0.006	−0.008
2000	0.048	0.052	0.012	0.010
2004	0.111	0.110	0.023	0.031
2006	0.119	0.119	−0.033	−0.030
2009	0.070	0.065	0.008	0.006
2011	0.030	0.032	−0.025	−0.024

In addition, to find out whether the new subjects of each wave would affect signs of concentration index of tested hypertension prevalence in total sample, we also conducted the Cs of baseline subjects in each wave and compared them with Cs of total population. The results are shown in the [Additional file 5: Table S5]. It is apparent that the signs of Cs of baseline subjects in each wave were generally consistent with signs of total population and the 95% CIs overlap in each year.

## Discussion

### The prevalence of self-reported hypertension and tested hypertension

We computed separately self-reported prevalence and tested prevalence from 1991 to 2011 in this article. Consistent with those studies, we find that the prevalence of both self-reported hypertension and tested hypertension have rapidly increased from 1991 to 2011 in rural China. This rapid increase may be because of the change of health behaviors in rural China. Overweight rose from 15.56 to 32.49%, and obesity raised from 3.04 to 12.05%. The role that obesity plays in hypertension prevalence has already been discussed in other literatures [41]. Our study emphasizes on this opinion and verifies that obesity in China has a rapid grow in the past decades. The population with tested hypertension was always significantly larger than that with self-reported hypertension [40]. The rise of self-reported prevalence is from 2.72 to 13.2% from 1991 to 2011, while the tested is from 11.01 to 25.05%. However, compared with prior studies, we found a lower prevalence of both self-reported hypertension and tested hypertension in rural China and a possible reason is that the new subjects of each year may drag the prevalence rate down. One study found that the prevalence of self-reported hypertension and tested hypertension in 2009 is 12.6 and 29.6% respectively [42], while the prevalence in our study is 9.46 and 21.07% respectively. Another study on rural resident aged 35–74 indicates that hypertension prevalence increased by 20% from 1991 to 2011 [40], however, in our study self-reported prevalence and tested prevalence increased by 6.52 and 4.25% respectively. This difference suggests that the growth of hypertension prevalence is lower in younger people than in elder people.

Our study also indicates that the prevalence rate of tested hypertension is nearly twice as that of self-reported hypertension; the ratio is lower than previous findings using a national survey [42]. A potential reason is that our results are from rural areas where basic public health services are less developed compare with urban areas and thus the ratio between tested hypertension and self-reported hypertension is a bit less than nationwide area. In other literatures, some researchers did not adopt the definitions of self-reported hypertension and tested hypertension but conducted research on hypertension prevalence and awareness. In a sense, the awareness of hypertension can describe self-reported prevalence.

Based on the uneven access and utilization of health resources, both our findings and prior findings indicate that different measurements of hypertension prevalence vary significantly [43]. Using self-reported hypertension measures implies substantial bias against the real prevalence of hypertension, and thus, the findings based on self-reported measures can be expected to mislead the government’s policy. The deviation caused by self-reported measures exists in many countries and is supposed to be higher in low-income and middle-income countries such as China [44].

### The main determinants of self-reported hypertension and tested hypertension

Our study indicates that age, BMI, region and marital status are all risk factors for hypertension, which is consistent with prior studies. For instance, in 2011, aging 60 and above shares more than 150% in the contribution to the concentration indexes of both self-reported prevalence and tested prevalence. BMI and region have significant impacts on hypertension prevalence. Some studies also suggested that income level could have an impact on hypertension prevalence [45], but in our study the impact of income level on the self-reported hypertension and the tested hypertension is respectively insignificant and negative. While many studies suggested that higher education level could reduce the probability of hypertension prevalence [8], our study shows a conflicting result that education level can strikingly affect tested hypertension prevalence, but not self-reported hypertension. The disparity may reflect education can help to improve individuals’ health consciousness, which in turn affects the actual control of blood pressure. Additionally, we find people having physical examinations in the past 4 weeks are more likely to get self-reported hypertension but unexpectedly, this result is not so significant for tested hypertension. A possible explanation is that those people who take physical examinations usually have more chances to be diagnosed by doctors and therefore it appears that they have more probability to have self-reported hypertension. More efforts should be put into equalizing basic public health services especially popularizing physical examination as it plays an important role in hypertension awareness and control.

### The equity of self-reported hypertension and tested hypertension

There are both similarities and differences between the findings of our study and prior studies. Some of them have proved that not only hypertension, but also some other chronic diseases are inequitable and pro-poor, such as diabetes and heart diseases [8]. We find that this is also true for tested hypertension. It is generally considered that the poor possess less health resources than the rich and thus suffer a worse health status. However, in our findings, the concentration index of self-reported prevalence is positive, for example, 0.118 [95%CI (0.023,0.213)] in 1991 and 0.065 [95%CI (0.026,0.104)] in 2009, which means that self-reported prevalence concentrates on the rich from 1991 to 2011, while the Cs of tested prevalence were negative in some years. This conflicting result might be due to the ignorance of differences in two measurements of hypertension in prior studies. The significant disparities between the rich and the poor, in the access to and utilization of basic health services are taken into consideration for our study. Some researchers shared the same reasoning – it is evident that people in states that provide more education and better medical and health facilities are in a better position to be diagnosed and aware of their own particular illnesses than the people in states providing less education and worse medical and health facilities, where there is less awareness of treatable conditions [46].

Combining the equity with the prevalence of the two hypertensions, we can find that the income-related inequality of self-reported prevalence is pro-rich as its concentration indexes and horizontal-inequity indexes are always positive in 8 years. In addition, although the tested prevalence has rapidly increased from 1991 to 2011, the concentration indexes are always close to 0. This indicates that the prevalence of tested hypertension is less related to income level. This reveals the access to health resources and services in rural China are pro-rich, even though China is strongly pushing the equalization of basic public health services. There is no doubt that China’s basic public health services are getting increasingly equal to everyone and the quantity of health funds has been devoted into basic services such as hypertension screening ever since the equalization policy of basic public health services was carried out. Nevertheless, there still is a gap which cannot be ignored between the poor and rich in health accessibility and utilization. The imbalanced accessibility and utilization of health services might be the cause of the contradictory result in this study and this finding should raise our attention to put more effort into health service equity. Hence, if we focus on self-reported hypertension solely, a biased conclusion or policy will probably come out. Additionally, for the realization of the right to maintain health, China stressed the importance of universal health coverage (UHC) [47]. As a result, in 2011, about 95.7% of the Chinese population is covered by three main health insurances. In spite of the achievement of UHC, the access to health services and resources is not sufficient yet. Researchers conducted a study to assess the effective coverage of health insurance to explore whether the expansion of health insurance can improve health status [48], while the insufficient access to health services and resources indicated in our study may reveal that the effective coverage of health insurance in rural China is still low. The government may implement relevant policies to promote effective coverage of health services, not only crude coverage.

China has implemented a series of policies to improve the access to health care in rural areas. But current policies cannot sufficiently meet the challenges of promoting effective coverage of health care. Developing social capital in rural area can be a potential solution to promote the management of chronic diseases [49], but systematic measurements have not been well documented yet. It is advisable to establish free health management model in rural China to solve pro-rich access problem. Currently, free physical examination can be received by rural residents. But for those who have been monitored for chronic diseases, free management measures are not provided except outpatient reimbursement for certain kinds of diseases [50]. The feasibility of providing national essential medicines for free in chronic diseases among the elderly has been studied and researchers found that it could be financially guaranteed but a further systematic study is needed [51].

## Conclusions

Our study indicates that there are deviating results between self-reported hypertension and tested hypertension both in prevalence and equity. There are several suggestions proposed by our research. First of all, more efforts should be put into raising the health status of the poor, especially in equal access to health services. Furthermore, adopting self-reported measures solely in research may mislead our policy-making and thus symptom-based measures such as tested hypertension should be adopted more widely in empirical studies.

We acknowledge some limitations in our analysis. The most recent year of our study is 2011 and we have no access to more recent data, thus analysis using data of more recent years are necessary for further study. Another limitation is that in some earlier years of our study such as 1991 and 1993, several independent variables had too few observations and thus were excluded in our regression model. This may result in a minor error of horizontal-inequity index. Lastly, difference between self-reported hypertension prevalence of baseline subjects and new subjects has been proved that have no significance in most years and the Cs of baseline subjects and total subjects have no significant difference, but still there are some potential factors that may affect prevalence and concentration index, although we have tried our best to solve the problem.

## Additional files

Additional file 1: **Table S1.** Subjects and new subjects of each year. (PDF 56 kb)

Additional file 2: **Figure S1.** Age-adjusted prevalence of self-reported hypertension and tested hypertension. (PDF 49 kb)

Additional file 3: **Table S2.** The Chi-Square test of prevalence of baseline subjects and new subjects. (PDF 68 kb)

Additional file 4: **Table S3.** The regression effect of 1991, 1993 and 1997. **Table S4.** the regression effect of 2000, 2004, 2006 and 2009. (PDF 238 kb)

Additional file 5: **Table S5.** Concentration index of tested hypertension prevalence. (PDF 63 kb)

## Data Availability

The datasets generated and analyzed during the current study were derived from the China Health and Nutrition Survey (CHNS) conducted in 2011. They are opened to everyone. Researchers who want to use these data can visit http://www.cpc.unc.edu/projects/china).

## Abbreviations

95% CI: 95% Confidence interval
BMI: Body mass index
C: Concentration Index
DBP: Diastolic blood pressure
HI: Horizontal-inequity Index
SBP: Systolic blood pressure
