# Supplementary material for: Prevalence and income-related equity in hypertension in rural China from 1991 to 2011: differences between self-reported and tested measures
Source: BMC Health Serv Res. 2019 Jul 1;19:437. doi: 10.1186/s12913-019-4289-5 (PMC6604163; doi:10.1186/s12913-019-4289-5)
Supplement: Supplementary file 1 — Table S1. Subjects and new subjects of each year. (PDF 56 kb) [file 12913_2019_4289_MOESM1_ESM.pdf]

Table S1 Subjects and new subjects of each year

Year	Subjects	New subjects compared to last year (%)
1991	11119	-
1993	10828	932 (8.6)
1997	11891	4037 (33.95)
2000	13324	3575 (26.83)
2004	13194	2689 (20.38)
2006	15922	4297 (26.99)
2009	16313	3316 (20.33)
2011	19722	5754 (29.18)
