# Supplementary figures and images for: Prevalence and income-related equity in hypertension in rural China from 1991 to 2011: differences between self-reported and tested measures
Source: BMC Health Serv Res. 2019 Jul 1;19:437. doi: 10.1186/s12913-019-4289-5 (PMC6604163; doi:10.1186/s12913-019-4289-5)

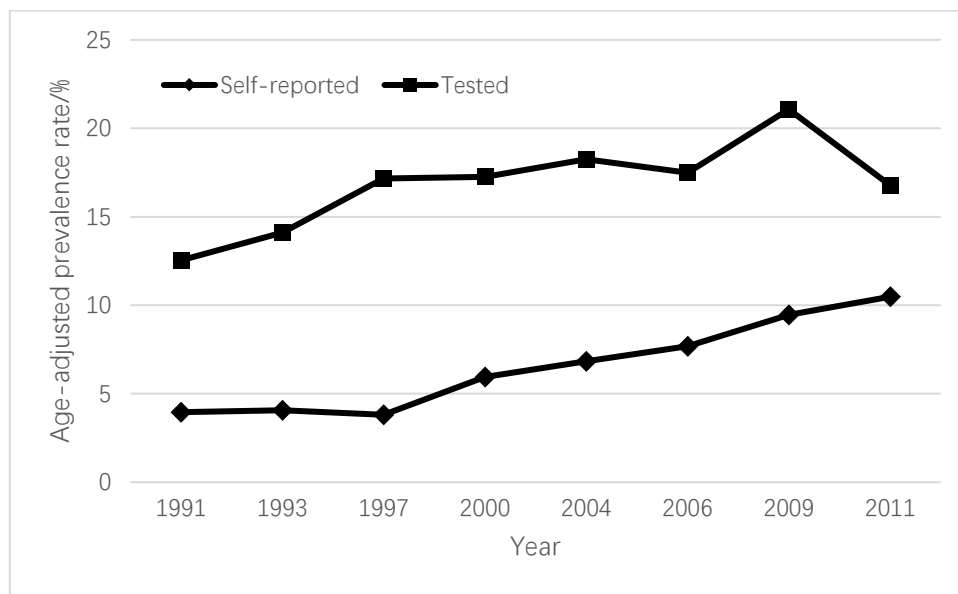

Supplement: Supplementary file 2 — Figure S1. Age-adjusted prevalence of self-reported hypertension and tested hypertension. (PDF 49 kb) [file 12913_2019_4289_MOESM2_ESM.pdf]
