# Supplementary material for: Prevalence and income-related equity in hypertension in rural China from 1991 to 2011: differences between self-reported and tested measures
Source: BMC Health Serv Res. 2019 Jul 1;19:437. doi: 10.1186/s12913-019-4289-5 (PMC6604163; doi:10.1186/s12913-019-4289-5)
Supplement: Supplementary file 3 — Table S2. The Chi-Square test of prevalence of baseline subjects and new subjects. (PDF 68 kb) [file 12913_2019_4289_MOESM3_ESM.pdf]

Table S2 The Chi2-Square test of prevalence of baseline subjects and new subjects

Year	*p* values
1991	-
1993	0.654
1997	0.002
2000	0.213
2004	0.209
2006	0.006
2009	<0.001
2011	0.699
