# Supplementary material for: Prevalence and income-related equity in hypertension in rural China from 1991 to 2011: differences between self-reported and tested measures
Source: BMC Health Serv Res. 2019 Jul 1;19:437. doi: 10.1186/s12913-019-4289-5 (PMC6604163; doi:10.1186/s12913-019-4289-5)
Supplement: Supplementary file 4 — Table S3. The regression effect of 1991, 1993 and 1997. Table S4. the regression effect of 2000, 2004, 2006 and 2009. (PDF 238 kb) [file 12913_2019_4289_MOESM4_ESM.pdf]

	1991						1993						1997					
	Self-reported hypertension			Tested hypertension			Self-reported hypertension			Tested hypertension			Self-reported hypertension			Tested hypertension		
	dy/dx	C	%	*dy/dx*	C	%	*dy/dx*	C	%	*dy/dx*	C	%	*dy/dx*	C	%	*dy/dx*	C	%
The poor	-0.0002	-0.400	0.57	-0.001	-0.400	0.9	0.003	-0.400	-5.96	-0.016	-0.400	-352.30	-0.005*	-0.400	25.17	-0.016	-0.400	-101.40
The middle	-0.001	0.002	0.01	0.016	0.002	0.1	0.008	0.0002	0.00	-0.014	0.0001	1.33	-0.003	0.001	-0.02	-0.029*	0.001	0.25
The richer	0.004	0.401	9.00	0.017	0.401	14.46	0.016***	0.400	30.06	-0.007	0.400	145.72	-0.004	0.400	-22.56	-0.032**	0.400	204.44
The richest	0.003	0.800	15.16	0.020	0.800	32.61	0.014**	0.800	49.33	-0.004	0.800	179.47	-0.0005	0.800	-4.55	-0.021	0.800	265.18
46~59	0.025***	0.040	5.72	0.102***	0.040	7.65	0.036***	0.082	12.18	0.111***	0.082	-457.63	0.031***	0.096	38.20	0.130***	0.096	-202.78
60 and above	0.079***	-0.504	-19.33	0.323***	-0.050	-26.58	0.108***	-0.075	-30.31	0.276***	-0.075	949.07	0.100***	-0.095	-106.23	0.315***	-0.095	425.82
18.5≤BMI<24	0.004	-0.016	-1.34	0.024**	-0.016	-2.81	0.002	-0.018	-0.58	0.027*	-0.018	95.01	0.007*	-0.033	-9.44	0.058***	-0.033	101.86
24≤BMI<28	0.028***	0.104	14.06	0.166***	0.104	28.04	0.029***	0.082	9.40	0.103***	0.082	-414.58	0.034***	0.110	48.93	0.206***	0.110	-376.50
BMI≥28	0.119***	0.175	19.70	0.321***	0.175	17.80	0.139***	0.101	10.00	0.242***	0.101	-212.77	0.112***	0.120	43.10	0.373***	0.120	-181.84
Primary and junior high school	0.005	0.022	2.07	0.006	0.022	0.87	0.0001	0.025	0.04	0.004	0.025	-18.53	0.007**	0.007	1.77	-0.019	0.007	6.48
High school or technical secondary school	-0.004	0.117	-1.87	-0.022	0.117	-3.58	-0.003	0.112	-1.06	-0.022	0.112	95.34	-0.004	107	-4.67	-0.046	0.107	65.51
Junior college and above	0.011	0.224	1.82	0.003	0.224	2.0	0.003	0.163	0.30	-0.065*	0.016	76.60				-0.011	0.140	3.46
Married	0.016**	0.005	2.03	0.031**	0.005	1.32	0.005	-0.001	-0.12	0.043***	-0.001	12.62	0.009*	0.0004	0.17	0.027	0.0004	-0.70
Other status	0.050**	-0.079	-8.21	0.068***	-0.079	-3.74	0.006	-0.085	-0.85	0.110***	-0.085	183.01	0.028**	-0.103	-12.92	0.124***	-0.103	72.38
The middle region	-0.001	-0.017	0.21	-0.018**	-0.017	1.67	0.007**	-0.050	-4.19	-0.022**	-0.050	-162.71	0.004	-0.046	-6.66	-0.026**	-0.046	-58.72
The western region	-0.008	-0.223	7.40	-0.027**	-0.223	8.63	-0.009***	-0.113	3.24	-0.058***	-0.113	-267.20	-0.005	-0.205	9.44	-0.083***	-0.205	-197.90
Smoking	-0.005	-0.006	0.31	-0.008	-0.006	1.66	0.001	0.002	0.02	0.011	0.002	-2.51	0.0002	-0.027	-0.13	0.013	-0.027	9.39
Female	-0.001	-0.003	0.03	-0.034***	-0.003	0.45	-0.002	-0.008	0.23	-0.037***	-0.008	-43.60	0.002	-0.002	-0.12	-0.057***	-0.002	-5.32
Drinking	0.007	0.103	2.41	0.046***	0.103	5.53	0.002	0.142	0.65	-0.0004	0.033	1.26	-0.003	0.096	-2.29	0.013	0.096	-13.29
Having physical examination	0.060*	0.220	4.21	0.194***	0.220	4.60				-0.030	0.211	16.39	0.093***	0.092	4.86	0.021	0.092	-1.38
N		5009			5278			5122			5044			5307			5250	
R^2		0.1501			0.1919			0.1855			0.1335			0.1953			0.1487	

*, **, ***: significantly different from zero at the 0.1, 0.05 and 0.01 level, respectively

	2000						2004						2006						2009					
	Self-reported			Tested			Self-reported			Tested			Self-reported			Tested			Self-reported			Tested		
	dy/dx	C	%	*dy/dx*	C	%	*dy/dx*	C	%	*dy/dx*	C	%	*dy/dx*	C	%	*dy/dx*	C	%	*dy/dx*	C	%	*dy/dx*	C	%
The poor	-0.009	-0.400	27.23	-0.042**	-0.400	175.42	0.003	-0.400	-3.22	0.021	-0.399	-26.55	-0.002	-0.400	1.38	-0.014	-0.400	-18.58	-0.012	-0.400	13.02	-0.026	-0.400	130.58
The middle	-0.007	0.0004	0.00	-0.004	0.0005	0.00	0.017**	0.001	0.00	0.028*	0.001	0.01	-0.001	0.001	0.000	-0.022	0.001	0.04	-0.009	0.000	0.00	-0.021	0.000	-0.04
The richer	-0.006	0.400	-16.12	-0.030*	0.400	-126.85	0.001	0.400	1.42	0.004	0.400	4.75	0.006	0.401	5.00	-0.041**	0.401	53.22	-0.004	0.400	-4.80	-0.012	0.400	-60.83
The richest	-0.004	0.800	-24.81	-0.019	0.800	-158.74	0.015*	0.800	30.85	0.024	0.800	59.93	0.030***	0.800	50.62	-0.008	0.800	21.99	0.019*	0.800	43.58	-0.035*	0.800	-352.78
46~59	0.056***	0.010	47.31	0.119***	0.010	142.17	0.091***	0.040	11.95	0.149***	0.040	23.65	0.090***	0.040	8.13	0.118***	0.040	-16.45	0.119***	0.058	21.99	0.193***	0.058	162.23
60 and above	0.172***	-0.106	-120.23	0.358***	-0.106	-357.69	0.211***	-0.062	-35.17	0.328***	-0.062	-66.27	0.224***	-0.120	-54.47	0.262***	-0.120	97.68	0.252***	-0.072	-53.21	0.334***	-0.072	-321.36
18.5≤BMI<24	0.012	-0.043	-11.56	0.051**	-0.043	-69.58	0.013	-0.037	-3.55	-0.006	-0.037	1.97	0.020*	-0.032	-3.78	0.064***	-0.032	19.11	0.055***	-0.025	-10.61	0.074***	-0.025	-64.45
24≤BMI<28	0.065***	0.091	55.07	0.187***	0.091	225.25	0.062***	0.080	18.00	0.138***	0.080	48.21	0.067***	0.067	13.73	0.180***	0.067	-56.36	0.143***	0.048	28.66	0.194***	0.048	177.52
BMI≥28	0.174***	0.117	50.64	0.416***	0.112	172.96	0.183***	0.106	20.54	0.312***	0.106	42.23	0.217***	0.082	15.86	0.389***	0.082	-43.70	0.289***	0.065	25.50	0.361***	0.065	144.69
Primary and junior high school	0.008	-0.008	-1.46	-0.019	-0.008	5.03	0.005	-0.032	-1.40	-0.027**	-0.032	8.55	0.011*	-0.034	-2.19	-0.066***	-0.034	-21.14	0.005	-0.026	-1.02	-0.009	-0.026	9.38
High school or technical secondary school	0.001	0.139	0.69	-0.009	0.139	-11.26	0.005	0.130	1.74	-0.030	0.130	-12.39	0.018*	0.152	6.00	-0.097***	0.152	50.51	-0.008	0.133	-2.86	-0.046**	0.133	-75.51
Junior college and above	-0.005	0.240	-1.85	0.025	0.240	12.10	-0.027*	0.259	-4.41	-0.043	0.259	-8.70	0.033	0.256	5.82	-0.128***	0.256	35.10	-0.029	0.226	-5.63	-0.115***	0.226	-102.45
Married	0.015	0.004	1.73	0.036*	0.004	6.06	0.006	0.006	0.25	0.006	0.006	0.40	0.048**	0.004	1.77	0.071**	0.004	-4.05	0.066**	0.012	9.49	0.060*	0.012	39.22
Other status	0.026	-0.104	-6.97	0.068**	-0.104	-26.00	0.017	-0.091	-1.77	0.060**	-0.091	-7.48	0.174**	-0.119	-20.49	0.136***	-0.119	24.62	0.195***	-0.133	-37.33	0.141***	-0.133	-122.50
The middle region	-0.007	-0.109	15.6	-0.024**	-0.109	74.34	-0.011*	-0.092	7.22	0.001	-0.092	-1.03	-0.03	-0.068	1.21	-0.009	-0.068	-5.75	-0.013*	-0.094	9.37	-0.061***	-0.094	206.80
The western region	-0.023***	-0.150	15.87	-0.074***	-0.150	72.97	-0.014*	-0.190	4.48	-0.045***	-0.190	16.82	-0.018**	-0.132	3.45	-0.061***	-0.132	-18.35	-0.033***	-0.125	7.83	-0.092***	-0.125	98.32
Smoking	-0.006	-0.031	2.00	0.028	-0.031	-14.32	-0.002	0.005	0.00	-0.011	0.005	-0.28	0.004	0.018	0.25	-0.002	0.018	0.23	0.010	-0.001	-0.04	-0.019	-0.001	0.36
Female	0.007	-0.001	0.00	-0.027**	-0.001	0.50	0.007	-0.011	-0.54	-0.080***	-0.011	7.12	0.008	-0.017	-0.77	-0.066***	-0.017	-9.73	0.015*	-0.018	-1.95	-0.074***	-0.018	45.37
Drinking	0.004	0.079	1.44	0.034**	0.079	15.92	0.011	0.069	1.10	0.028*	0.069	3.41	-0.002	0.075	-0.16	0.038**	0.075	-4.14	-0.011	0.197	-2.60	0.010	0.197	10.17
Having physical examination	0.070*	0.188	4.00	0.038	0.188	3.07	0.094***	0.310	13.11	0.069**	0.310	11.66	0.046***	0.104	1.69	0.023	0.104	-1.33	0.110***	0.169	10.44	0.059*	0.169	25.45
N		5205			5248			6118			5910			6042			5909			6396			5594	
R^2		0.1750			0.1481			0.1857			0.1387			0.1871			0.1313			0.1800			0.1324	

*, **, ***: significantly different from zero at the 0.1, 0.05 and 0.01 level, respectively
