# Supplementary material for: Prevalence and income-related equity in hypertension in rural China from 1991 to 2011: differences between self-reported and tested measures
Source: BMC Health Serv Res. 2019 Jul 1;19:437. doi: 10.1186/s12913-019-4289-5 (PMC6604163; doi:10.1186/s12913-019-4289-5)
Supplement: Supplementary file 5 — Table S5. Concentration index of tested hypertension prevalence. (PDF 63 kb) [file 12913_2019_4289_MOESM5_ESM.pdf]

Table S5 Concentration index of tested hypertension prevalence

| Year | Baseline subjects | | | Total subjects | | |
|---|---|---|---|---|---|---|
| | *C* | 95% CI | | *C* | 95% CI | |
| 1991 | 0.088 | 0.024 | 0.112 | 0.088 | 0.024 | 0.112 |
| 1993 | -0.032 | -0.066 | 0.003 | -0.003 | -0.004 | 0.037 |
| 1997 | -0.068 | -0.109 | -0.027 | -0.008 | -0.060 | 0.005 |
| 2000 | -0.010 | -0.041 | 0.022 | 0.010 | -0.032 | 0.028 |
| 2004 | 0.040 | 0.010 | 0.070 | 0.031 | -0.023 | 0.034 |
| 2006 | 0.0002 | -0.030 | 0.030 | -0.030 | -0.061 | -0.004 |
| 2009 | 0.040 | 0.011 | 0.070 | 0.006 | -0.035 | 0.017 |
| 2011 | -0.020 | -0.053 | 0.135 | -0.024 | -0.038 | 0.009 |
